# Synthesis of Finely Controllable Sizes of Au Nanoparticles on a Silica Template and Their Nanozyme Properties

**DOI:** 10.3390/ijms221910382

**Published:** 2021-09-26

**Authors:** Bomi Seong, Jaehi Kim, Wooyeon Kim, Sang Hun Lee, Xuan-Hung Pham, Bong-Hyun Jun

**Affiliations:** 1 Department of Bioscience and Biotechnology, Konkuk University, Seoul 05029, Korea; iambomi33@konkuk.ac.kr (B.S.); susia45@gmail.com (J.K.); buzinga5842@konkuk.ac.kr (W.K.); 2 Department of Chemical and Biological Engineering, Hanbat National University, Daejeon 34158, Korea; sanghunlee@hanbat.ac.kr

**Keywords:** gold nanoparticles, nanoparticle size, gold-assembled silica nanostructures, local surface plasmon resonance, nanozyme, peroxidase-like activity, nanoparticle, nanosphere, aggregation, fine controllable size

## Abstract

The precise synthesis of fine-sized nanoparticles is critical for realizing the advantages of nanoparticles for various applications. We developed a technique for preparing finely controllable sizes of gold nanoparticles (Au NPs) on a silica template, using the seed-mediated growth and interval dropping methods. These Au NPs, embedded on silica nanospheres (SiO_2_@Au NPs), possess peroxidase-like activity as nanozymes and have several advantages over other nanoparticle-based nanozymes. We confirmed their peroxidase activity; in addition, factors affecting the activity were investigated by varying the reaction conditions, such as concentrations of tetramethyl benzidine and H_2_O_2_, pH, particle amount, reaction time, and termination time. We found that SiO_2_@Au NPs are highly stable under long-term storage and reusable for five cycles. Our study, therefore, provides a novel method for controlling the properties of nanoparticles and for developing nanoparticle-based nanozymes.

## 1. Introduction

Enzymes are biocatalysts that play an important role in living systems. However, they are expensive, difficult to store, laborious to produce, and are easily denatured in external environments from varying temperature, pH, and chemical stressors [1,2]. These drawbacks critically limit their practical uses [3].

To overcome the above limitations, nanozymes have been developed as a new alternative to enzymes [1,4]. Nanozymes are nanomaterials that possess an intrinsic enzyme-like activity and have advantages such as stability in external environments, reasonable costs, and good catalytic activity [1,2,5,6,7,8,9].

Since the discovery of the unique peroxidase-like activity of Fe_3_O_4_ magnetic nanoparticles (NPs) by Yan’s group in 2007, numerous researchers around the world have gained an interest in nanozymes made of metal nanomaterials [10]. Metal nanomaterials, including gold (Au) NPs, platinum NPs, iron oxide NPs, cerium oxide NPs, manganese oxide NPs, and copper oxide NPs, began to be used as nanozymes [5,6,10,11,12,13,14,15]. Most of them have enzyme-like activities, such as those of peroxidases, catalases, oxidases, and superoxide oxidases [1,2,3,5,6,10,14,15,16,17,18,19,20,21,22].

Among the various metal nanomaterials, Au NPs have attracted the most attention because of their outstanding catalytic properties and advantages [23,24,25,26,27,28,29,30,31,32,33,34,35]. Au NPs can be synthesized easily and are stable [36,37]. In addition, the physical/chemical properties of Au NPs can be controlled by controlling their size and shape [24,26,33,35]. These NPs are also highly biocompatible and are easy to functionalize [24,30,35,38,39,40,41,42]. However, gold is not cheap, and the catalytic reactions depend on the surface area of the NPs. Fine-sized NPs can be cost-effective, but they are difficult to separate for reuse [1,3,8,9,10,17,43,44]. The development of a structure that uses small amounts of gold to acquire a large surface area, while still being easily separated, might prove to be a highly useful material in the field of nanozymes. A nanostructure where Au NPs are assembled onto a silica (SiO_2_) sphere core was developed by the Halas group in 1998 [45]. The chemical and optical properties of the Au NP-embedded SiO_2_ structure can be changed by simply controlling the diameter of the core and layered nanoparticles [46,47,48,49,50,51]. The NP-embedded SiO_2_ nanostructures are also cost-effective since only small amounts of expensive Au NPs are embedded onto the silica core, and they are easily separated from the reaction solution with the SiO_2_ core.

Au NP-assembled SiO_2_ nanostructures have been investigated in various fields [50,52,53,54,55,56] and have been found to have many merits due to the combined properties of Au NPs and of the silica core, simultaneously utilizing the outstanding and unique features of Au NPs and the inert and versatile feature of SiO_2_ [52,54,55,57]. In addition, the absorbance spectra of the nanostructures can be tuned across the visible and infrared regions by controlling the size of the Au NPs [38,39,58,59]. Due to these properties, the Au NP-assembled SiO_2_ nanostructures have broad applications. While there are many possible approaches for the control of the size and density of Au NPs on a template surface, a method of synthesis for those nanostructures has not yet been established [46,48,49,50,58,59]. For this reason, the low density and non-uniform morphology of Au NPs on nanostructures remain a considerable challenge [46,48,49,50]. There is, therefore, a need for the development of an improved method for preparing Au-NP-assembled silica nanostructures.

Our group recently developed SiO_2_@Au nanostructures in which Au NPs were densely immobilized on the surface of a SiO_2_ nanosphere [60]. For this nanostructure, the SiO_2_ nanosphere was used as a template, and the Au NPs were uniformly and densely introduced on it, using the seed-mediated growth method. SiO_2_@Au nanostructures have enhanced separation and re-dispersion properties and are more stable during surface modification than Au NPs. Moreover, they have shown potential as effective nanozymes. In this study, besides introducing dense and uniform Au NPs onto the SiO_2_ nanospheres, we also developed a facile method to very precisely control the size of Au NPs on the SiO_2_ surface, and we investigated their optical and catalytic characteristics by controlling the size of the Au NPs. Furthermore, various factors affecting the peroxidase-like activity of SiO_2_@Au NPs were also studied.

## 2. Results and Discussion

### 2.1. Preparation of Size-Controlled Au NPs-Assembled Silica Nanostructures (SiO_2_@Au NPs)

The SiO_2_@Au NPs were prepared by using seed-mediated growth synthesis, consisting of two steps: embedding Au seeds on the SiO_2_ surface, and growth of the Au NPs via the addition of an Au^3+^ precursor and reductant in intervals [45,52]. First, the Au seeds (~2.5 nm) were prepared by using an Au^3+^ precursor (HAuCl_4_) and tetrakis(hydroxymethyl)phosphonium chloride (THPC). Then, the Au seeds were incubated with aminated SiO_2_ nanospheres (~160 nm) to obtain Au-seeded SiO_2_ nanospheres, as previously reported [56,57,60,61,62]. On the Au-seeded SiO_2_ nanospheres, the reduction of the Au^3+^ precursor was directly conducted by using ascorbic acid (AA), which is a mild reducing agent, in the presence of polyvinylpyrrolidone (PVP) as a stabilizer. In these mildly reducing conditions, the progress of the growth stage is much slower than in strongly reducing conditions, making it easier to control the growth procedure [36]. A low concentration of the Au^3+^ precursor and AA were added onto the Au-seeded SiO_2_ in 5 min intervals until the desired concentrations for fine control over the size of the Au NPs are attained. The amounts are indicated in Table 1.

**Figure 1:**
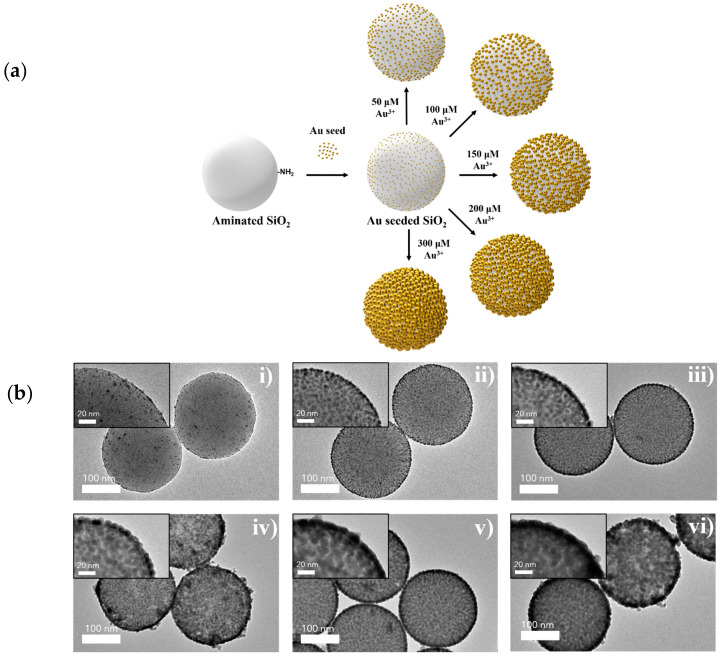
(**a**) Typical scheme of synthesis process of SiO_2_@Au nanoparticles. (**b**) The transmission electronic microscopy (TEM) images of gold-embedded silica nanospheres (SiO_2_@Au NPs) fabricated in various concentrations of Au^3+^: (**i**) 0 μM, (**ii**) 50 μM, (**iii**) 100 μM, (**iv**) 150 μM, (**v**) 200 μM, and (**vi**) 300 μM.

First, numerous small Au NPs (~2.5 nm) were attached throughout the SiO_2_ surface as seeds for the further growth of Au NPs. It is called SiO_2_@Au NP without the Au (III) precursor (0 µM), as shown in Figure 1b(i).

**Figure 2:**
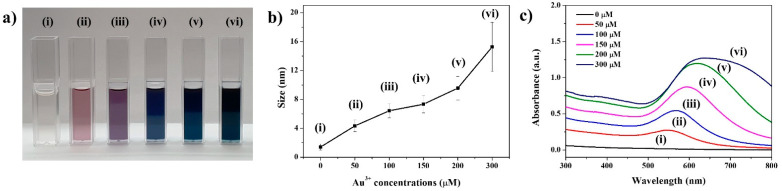
(**a**) Optical images and (**b**) size of Au NPs, and (**c**) UV–Vis absorption spectra of SiO_2_@Au NPs fabricated in various concentrations of Au^3+^ precursor: (i) 0 μM, (ii) 50 μM, (iii) 100 μM, (iv) 150 μM, (v) 200 μM, and (vi) 300 μM.

Subsequently, various concentrations of the Au^3+^ precursor were added to the Au-seeded SiO_2_ nanospheres at intervals; the size of the Au NPs grew larger as the Au^3+^ concentration was increased in Figure 1b(ii–vi). Moreover, the size of the Au NPs on the SiO_2_ nanospheres was precisely controlled with high levels of density and uniformity, which were confirmed clearly, as shown in Figure 1. At a high concentration of Au^3+^ (>200 µM), Au NPs on the SiO_2_ surface merged with each other and became one larger particle. When the size of the Au NPs increased, the color of the solution changed in the order of pale brown, then pink, purple, dark blue, and finally black (Figure 2a). These changes occurred depending on the size and shape of the NPs, due to their localized surface plasmon resonance (LSPR) [37,46,48]. When nanoparticles are close to one another, the absorption spectra of proximally located nanoparticles red-shift considerably from that of solitary particles [58,59]. Increasing the space between the particles reduces the shift [59]. Mie’s theory accounts for how increasing nanoparticle diameters induces the absorption spectra to red-shift by changing the electric surface charge density of the NPs [58]. The results of our study showed that the growth of Au NPs on SiO_2_@Au was controlled well by the color change of the particle suspension, transmission electron microscopy (TEM) images, and absorbance according to the abovementioned theories. 

The absorption spectra of SiO_2_ showed that the absorbance band was red-shifted and broadened upon an increase in the concentration of the Au^3+^ precursor, indicating the formation of larger Au NPs, which change the proximate interparticle distance (Figure 2b,c). In the absorbance band of SiO_2_@Au-NPs-treated 0 μM Au^3+^ precursor, no peak was observed due to the exceedingly tiny size of the Au NPs. On the other hand, 50, 100, 150, 200, and 300 Au^3+^-treated SiO_2_@Au showed peaks at 543, 571, 593, 619, and 632 nm respectively (Figure 2c). Moreover, the absorbance bandwidth broadened as the size of the Au NPs was increased.

### 2.2. Verification of the Peroxidase-like Activity of SiO_2_@Au NPs

**Figure 3:**
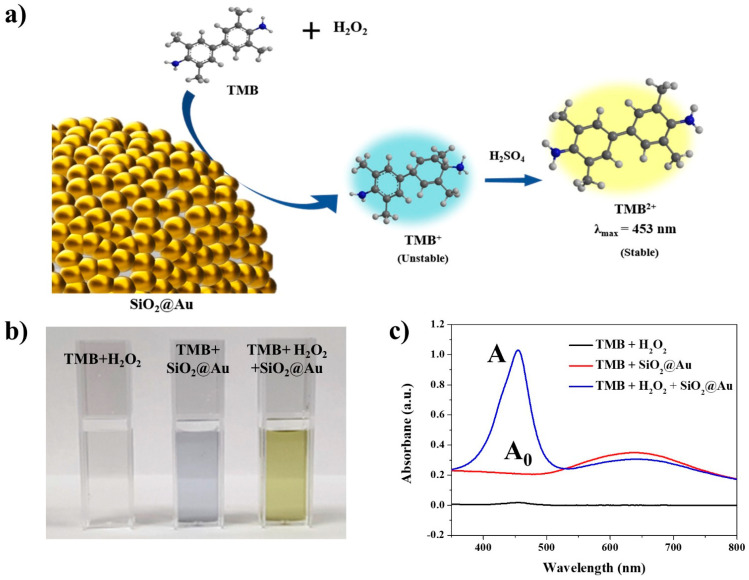
(**a**) Illustration of the peroxidase-like activity of the SiO_2_@Au NPs. (**b**) Optical image and (**c**) UV–Vis absorption spectra of tetramethylbenzidine (TMB) + H_2_O_2_, TMB + SiO_2_@Au NPs, and TMB + H_2_O_2_ + SiO_2_@Au NPs after termination with 1 M H_2_SO_4_ in the mixture of 1 mM TMB and 200 mM H_2_O_2_. The peak which appears at 453 nm wavelength originated from the oxidized TMB substrate.

The peroxidase-like activity of SiO_2_@Au NPs was evaluated through oxidation of a 3,3′,5,5′-tetramethylbenzidine (TMB) substrate. The TMB oxidation reaction involves the transfer of two electrons that each produce a clear color change. When the first electron is transferred to form TMB^+^ via oxidization of TMB, the TMB solution changes from colorless to blue. Since TMB^+^ is quite unstable in an acidic environment, it further oxidizes to TMB^2+^ when the second electron is transferred; TMB^2+^ is stable in acidic conditions, exhibiting a yellow color and a maximum absorption peak at 453 nm (Figure 3a) [63]. 

To confirm the peroxidase-like activity of the SiO_2_@Au NPs, TMB + H_2_O_2_, TMB + SiO_2_@Au NPs, and TMB + H_2_O_2_ + SiO_2_@Au NPs were prepared in a pH 4 buffer for the peroxidase assay. The TMB + H_2_O_2_ solution was colorless, and an absorbance peak at 453 nm did not appear, as shown in Figure 3b,c. This result indicated that peroxidase-like activity did not occur in the absence of SiO_2_@Au. Next, the color of the TMB + SiO_2_@Au NP sample was entirely on account of the SiO_2_@Au NPs, displaying an absorbance band with a maximum peak at 630 nm. However, a yellow solution and an absorbance band at 453 nm were observed in the TMB + H_2_O_2_ + SiO_2_@Au NPs sample. These results showed that SiO_2_@Au NPs catalyzed TMB oxidation in the presence of H_2_O_2_, indicating that a peroxidase-like reaction occurred due to the peroxidase-mimicking property of SiO_2_@Au NPs.

### 2.3. The Peroxidase-like Activity Depends on the Size of the Au NPs of the SiO_2_@Au NPs

**Figure 4:**
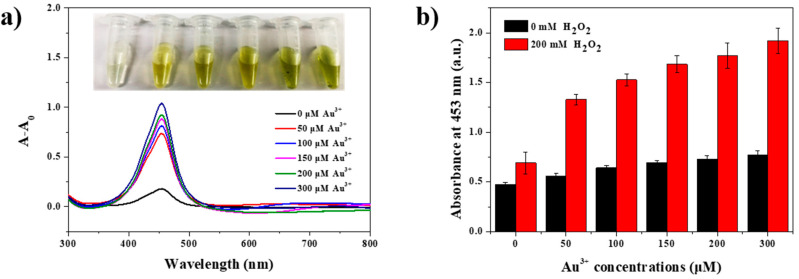
(**a**) UV–Vis absorption spectra of SiO_2_@Au NPs in the presence of TMB without H_2_O_2_ (A_0_) and with H_2_O_2_ (A). (**b**) Absorbance plots of 1 mM TMB and 200 mM H_2_O_2_ in the presence of various SiO_2_@Au NPs fabricated in different Au^3+^ concentrations in the range of 0 to 300 µM.

To investigate the correlation between the size of the Au NPs and the peroxidase-like activity of SiO_2_@Au NPs, various kinds of SiO_2_@Au NPs with different Au NPs sizes were prepared. The concentrations of the treated Au^3+^ precursors were 0, 50, 100, 150, 200, and 300 µM each, resulting in the formation of 1.4, 4.3, 6.4, 7.3, 9.5, and 15 nm diameter Au NPs on the SiO_2_@Au structures, respectively (Table 1 and Figure 2b). Each of these SiO_2_@Au NPs was subjected to the TMB assay to estimate their peroxidase-like activity. An absorbance peak at 453 nm was observed in the UV–Vis absorption spectra of all samples, indicating that all of the SiO_2_@Au NPs had peroxidase-like activity, irrespective of the size of the Au NPs (Supplementary Materials Figure S1). However, the SiO_2_@Au NPs produced without any Au^3+^ precursor treatment showed relatively very weak peroxidase-like activity. This may be because the size of the Au NPs on the silica core was too small; there were a large number of vacant spaces on the SiO_2_@Au NPs with the given number of Au NPs, providing an insufficient surface area for the reaction between the Au NPs and reactants. On the other hand, SiO_2_@Au NPs treated with more than 50 µM Au^3+^ precursor showed high peroxidase-like activity. The size of the Au NPs was rapidly increased when concentrations of Au^3+^ precursor exceeded 50 µM, as shown in the TEM images (Figure 1b(ii). As the size of the Au NPs on the SiO_2_@Au NPs was increased, the surface area which can react with reactants also increased. Therefore, the peroxidase-like activity of the SiO_2_@Au NPs was increased as the concentrations of the Au^3+^ precursor were increased (Figure 4a,b). Even though the size of the Au NPs grew as the concentration of the Au^3+^ precursor increased, severe aggregation occurred immediately after the peroxidase reaction in the SiO_2_@Au was treated with over 200 µM of the Au^3+^ precursor. Since good dispersibility is an important factor for generating a constant and stable catalytic activity, 150 µM of Au^3+^ precursor-treated SiO_2_@Au NPs, which have high peroxidase-like activity without aggregation, was used in the subsequent experiments [64,65].

### 2.4. Effects of Reaction Conditions on the Peroxidase-like Activity of SiO_2_@Au NPs

**Figure 5:**
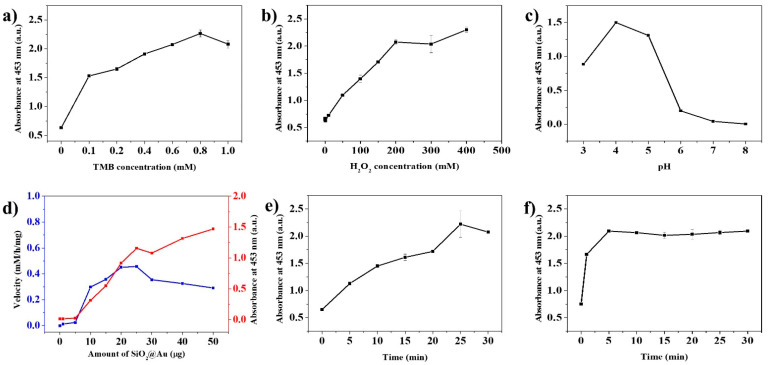
Effects of different reaction conditions on the peroxidase-like activity of SiO_2_@Au NPs in a mixture of TMB and H_2_O_2_: (**a**) TMB concentration, (**b**) H_2_O_2_ concentration, (**c**) pH value of the solution, (**d**) the velocity and amount of SiO_2_@Au, (**e**) reaction time, and (**f**) termination time.

It is known that the catalytic activity of nanozymes is affected by reaction conditions such as those associated with an enzyme [10,32,66,67,68,69]. For this reason, the effect of reaction conditions on the peroxidase-like activity on SiO_2_@Au NPs was investigated. The concentration of TMB and H_2_O_2_, pH of the buffer, the number of SiO_2_@Au NPs, reaction time, and termination time were considered in this study. For confirming the effects of TMB concentrations, the concentrations of TMB varied from 0 to 1.0 mM, while the other conditions were fixed in the peroxidase assay (Supplementary Materials Figure S2). The catalytic activity of SiO_2_@Au NPs increased until the concentrations of TMB were 0.8 mM; they then decreased at 1.0 mM, because the poor solubility of TMB in an aqueous buffer caused precipitation during the oxidation reaction (Figure 5a) [70]. To calculate the kinetic activities of SiO_2_@Au toward TMB concentration, various concentration of TMB in the range of 0.1 to 0.4 mM were prepared and then mixed to H_2_O_2_, and the absorbance were monitored every 10s. The absorbance at 200 s was used to calculate the Michaelis–Menten constants (K_m_) and the maximum reaction velocity (V_max_) in our study. The kinetic activities of SiO_2_@Au toward TMB concentration in the range of 0.1 to 0.4 mM TMB were plotted in Supplementary Materials Figure S3. A linear regression was found in the concentration of TMB from 0.1 to 0.4 mM. K_m_ were obtained by using Linewaerver–Burk plots. The apparent Michaelis constant K_m_ was calculated to be 0.060 mM and the maximum reaction velocity V_max_ was 2.3 × 10^−10^ M^−1^∙s^−1^. K_m_ of SiO_2_@Au was much lower than that of horseradish peroxidase enzyme, indicating that SiO_2_@Au has a higher affinity for TMB than horseradish peroxidase (K_m_ = 0.438 mM). Moreover, K_m_ of SiO_2_@Au is lower than those Au NPs (K_m_ = 0.123 mM), glucose-oxidase-conjugated Au-attached magnetic SiO_2_ microsphere (K_m_ = 0.208 mM), Au-NPs-decorated porous silica microsphere (K_m_ = 0.523 mM), Prussian-blue-decorated latex nanoparticle (K_m_ = 2.19 mM), MnO_2_ nanoparticles (K_m_ = 0.083 mM), and sulfate-latex-conjugated polyelectrolyte functionalized MnO_2_ NPs (K_m_ = 0.099 mM) [71,72,73,74,75]. 

In the case of the concentration of hydrogen peroxide, the catalytic activity of SiO_2_@Au NPs was increased steeply until reaching 200 mM H_2_O_2_ and decreased at a concentration of 300 mM H_2_O_2_ (Supplementary Materials Figure S4). Even though the activity again increased at 400 mM H_2_O_2_, the rate of increase was lower than 200 mM H_2_O_2_ (Figure 5b). Subsequently, various buffers with different pH were subjected to the peroxidase assay (Supplementary Materials Figure S5). The highest activity was shown under pH 4.0, at which H_2_O_2_ was more stable and TMB dissolved maximally (Figure 5c) [10,44,70,76,77,78]. The velocity of the catalytic reaction showed the highest value when 20 and 25 µg of SiO_2_@Au NPs were treated in the sample where the rest of the conditions were fixed (Figure 5d and Supplementary Materials Figure S6). About 25 min was required for the TMB^+^ oxidation and 5 min for the termination of the TMB^+^ oxidation to obtain stable results (Figure 5e,f).

### 2.5. Long-Term Stability and Reusability Test of SiO_2_@Au as Nanozyme

**Figure 6:**
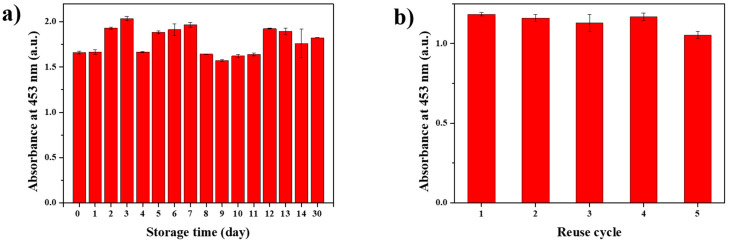
(**a**) Long-term stability of the peroxidase-like activity and (**b**) reusability of SiO_2_@Au NPs in a mixture of 0.6 mM TMB, 200 mM H_2_O_2_, and pH 4 buffer. The SiO_2_@Au NPs were stored in PBST (0.1%) at room temperature.

SiO_2_@Au NPs are substantially more advantageous over enzymes, owing to their long-term stability and reusability. Denaturation during storage and their on–off usage are the major defects when using enzymes in practice [1,2]. To verify the long-term stability of their peroxidase-like activity, the SiO_2_@Au NPs were examined by repeating the peroxidase assay every day, at the same time, for 14 days and on the 31st day after they were produced, keeping them under storage at 25 °C in the meantime (Figure 6a). The results showed that the peroxidase-like activity remained highly stable for at least 30 days after the fabrication of the SiO_2_@Au NPs. In sequence, the reusability of the SiO_2_@Au NPs as a nanozyme was evaluated through repeated peroxidase assays. Notably, the peroxidase-like activity of stored SiO_2_@Au until the fourth round was 98% of the first cycle level and mildly reduced at the fifth cycle to 89% of the first round. The reusability of SiO_2_@Au NPs was significantly high compared to a previous report on an Au NP-embedded silica nanostructure [79]. Moreover, SiO_2_@Au NPs remained 90% of catalytic activity, while SiO_2_@Au without the SiO_2_ core lost 60% of catalytic activity after five cycles of use (Supplementary Materials Figure S7). These results indicate that SiO_2_@Au NPs excel not only at being highly reusable but also at separating easily from the reaction mixture.

## 3. Materials and Methods

### 3.1. Chemicals and Reagents

Tetraethylorthosilicate (TEOS), tetrakis(hydroxymethyl)phosphonium chloride (THPC), chloroauric acid (HAuCl_4_), 3-aminopropyltriethoxysilane (APTS), AA, PVP (*M*_W_ 40,000), and TMB were purchased from Sigma-Aldrich (St. Louis, MO, USA). Ammonium hydroxide (NH_4_OH, 27%), ethyl alcohol (EtOH, 99.9%), sodium hydroxide (NaOH), and sulfuric acid (H_2_SO_4_) were purchased from Samchun (Seoul, Korea). Hydrogen peroxide (H_2_O_2_) was purchased from Daejung (Siheung, Gyeonggi-do, Korea). Phosphate buffer saline containing 0.1% Tween 20 (PBST, pH 7.4) was purchased from Dynebio (Seongnam, Gyeonggi-do, Korea).

### 3.2. Characterization

The transmission electron microscope (TEM) images of the samples were taken by using a JEM-F200 multi-purpose electron microscope (JEOL, Akishima, Tokyo, Japan) with a maximum accelerated voltage of 200 kV. The UV–Vis absorption spectra of the sample were measured by an Optizen POP UV/Vis spectrometer (Mecasys, Seoul, Korea). The centrifugation of samples was performed by using a microcentrifuge 1730R (LaboGene, Lyngen, Denmark).

### 3.3. Synthesis of Gold Nanoparticles (Au NPs) Assembled SiO_2_ Nanostructure (SiO_2_@Au NPs)

The SiO_2_@Au NPs were synthesized according to the previous report [60]. The colloidal Au was prepared by stirring 47.5 mL of water, 0.5 mL of 0.2 M NaOH, 12 μL of THPC, and 1 mL of 50 mM HAuCl_4_ for 1 h. Silica nanospheres (~160 nm) were prepared by using the modified Stöber method [80]. Briefly, 40 mL of EtOH, 1.6 mL TEOS, and 3 mL of NH_4_OH were allowed to react with each other for 20 h. The amino group was introduced to the surface of 2 mg SiO_2_ NPs by treating them with 62 μL of APTS. The aminated SiO_2_ NPs were incubated with colloidal Au (~2.5 nm) for 12 h. After several cycles of centrifugation of the mixture at 8500 rpm for 10 min, 2 mg of Au-seeded SiO_2_ NPs were obtained and dispersed in 2 mL of PVP solution (1 mg/mL of PVP in water). Subsequently, 200 μL of Au-seeded SiO_2_ NPs (1 mg/mL) suspension was added to 9.8 mL of PVP solution. Under stirring, 20 μL of 10 mM HAuCl_4_ solution (in water, Au^3+^ precursor) and 40 μL of AA solution, (10 mM AA in water, reducing agent) were added to the mixture in sequence. The reaction mixture was stirred for 5 min. To control the size of the Au NPs on the surface of the Au-seeded SiO_2_ nanospheres, 10 mM of Au^3+^ precursor and AA were added. Until the concentrations of Au^3+^ reached 50, 100, 150, 200, and 300 μM in the various mixtures, the same volumes of Au^3+^ precursor and AA were repeatedly added every 5 min. The SiO_2_@Au NPs were washed several times with centrifugation at 8500 rpm for 10 min. The washed SiO_2_@Au NPs were dispersed in 1 mL of 0.1% PBST solution to obtain a 0.2 mg/mL SiO_2_@Au NP suspension.

### 3.4. Peroxidase-like Activity of SiO_2_@Au

To verify the peroxidase-like activity of SiO_2_@Au NPs, 100 μL of TMB solution (10 mM in EtOH) and 100 μL of the various SiO_2_@Au NPs synthesized from 50, 100, 150, 200, and 300 μM Au^3+^, respectively, were added to 700 μL of pH 4 buffer. Then, freshly prepared 100 μL of H_2_O_2_ solution (2 M in pH 4 buffer) was added, and the mixture was incubated for 30 min at room temperature. To terminate the reaction, 500 μL of 1 M H_2_SO_4_ was added to each mixture and incubated for 10 min. The absorbance of the mixture at 350–800 nm was measured by using the UV–Vis spectrometer.

### 3.5. Peroxidase-like Activity of SiO_2_@Au in Various Reaction Conditions

#### 3.5.1. TMB Concentration

All assays of peroxidase-like activity were carried out in 1.5 mL Eppendorf tubes at room temperature. The TMB solutions were prepared in EtOH at various concentrations (1, 2, 4, 6, 8, and 10 mM, respectively). Then, 100 μL of each TMB solution, 100 μL of 2 M H_2_O_2_, and 100 μL of SiO_2_@Au (0.2 mg/mL) were added to 700 μL of pH 4 buffer. The final concentrations of TMB in the reaction mixture were 0.1, 0.2, 0.4, 0.6, 0.8, and 1.0 mM. After incubating the mixture for 30 min, 500 μL of 1 M H_2_SO_4_ was added to terminate the reaction. The absorbance of mixtures was measured by using a UV–Vis spectrometer.

#### 3.5.2. H_2_O_2_ Concentration

Various concentrations of H_2_O_2_ solutions (1, 2, 3, and 4 M) were prepared. Next, 100 μL of 6 mM TMB solution, 100 μL of each H_2_O_2_ solutions, and 100 μL of SiO_2_@Au (0.2 mg/mL) were added to 700 μL of pH 4 buffer. The final concentrations of H_2_O_2_ in the mixtures were 100, 200, 300, and 400 mM, respectively. After incubating the mixtures for 30 min at room temperature, we added 500 μL of 1 M H_2_SO_4_ to each to terminate the reaction.

#### 3.5.3. Buffer pH Value

Buffers of pH 3, 4, 5, 6, 7, and 8 were prepared. Next, 700 μL of each prepared buffer was added, followed by adding 100 μL of TMB solution, 100 μL of 2 M H_2_O_2_ solution, and 100 μL of SiO_2_@Au NPs (0.2 mg/mL). The mixtures were incubated for 30 min, and the reaction was terminated by using 500 μL of 1 M H_2_SO_4_.

#### 3.5.4. Amount of SiO_2_@Au NPs

All reagents, including 100 μL of 6 mM TMB solution, 100 μL 2 M H_2_O_2_ solution, and 700 μL pH 4 buffer, were added to tubes. Then 1, 5, 10 15, 20, 25, 30, 40, and 50 μg of SiO_2_@Au were dispersed in 100 μL PBST each and added to the mixtures, followed by incubation and termination.

#### 3.5.5. Reaction Time

To investigate the effect of reaction time on their peroxidase-like activity, the mixtures containing 100 μL of 6 mM TMB solution, 100 μL of SiO_2_@Au (0.2 mg/mL), and 100 μL of 2 M H_2_O_2_ solution were added to 700 μL of pH 4 buffer. Next, the mixtures were incubated for 0, 5, 10, 15, 20, 25, and 30 min respectively and terminated by using 1 M H_2_SO_4_.

#### 3.5.6. Termination Time

Mixtures including 700 μL of pH 4 buffer, 100 μL of 6 mM TMB solution, 100 μL of SiO_2_@Au (0.2 mg/mL), and 100 μL of 2 M H_2_O_2_ solution were prepared and incubated for 30 min. After adding 500 μL of 1 M H_2_SO_4_, we incubated each sample for 0, 5, 10, 15, 20, 25, and 30 min to terminate the reaction.

### 3.6. Long-Term Stability of Peroxidase-like Activity

To investigate the long-term stability of peroxidase-like activity of SiO_2_@Au, the experiment was repeated every day for 2 weeks and on the 31st day after their fabrication. The experimental procedures were conducted as follows: adding 100 μL of 6 M TMB solution, 100 μL of SiO_2_@Au (0.2 mg/mL), and 100 μL of 2 M H_2_O_2_ solution to 700 μL of pH 4 buffer; 30 min reaction time; 10 min for termination, using 500 μL of 1 M H_2_SO_4_.

### 3.7. Reusability as Nanozymes

To investigate the reusability of SiO_2_@Au, the peroxidase assay was performed according to the mentioned procedures. After the assay was performed, the SiO_2_@Au NPs were collected by using centrifugation at 10,000 rpm for 10 min, and the absorbance of the supernatant at 453 nm was measured by using a UV–Vis spectrometer. The peroxidase assay was repeated with the collected SiO_2_@Au NPs.

## 4. Conclusions

In summary, we successfully synthesized finely controllably sized Au NPs on the SiO_2_ nanosphere (SiO_2_@Au), using the seed-mediated growth method and interval dropping method under mild conditions. The effect of the size of Au NPs on the SiO_2_@Au NPs was confirmed by the TEM images, color changing of its suspension, and UV–Vis absorption spectra. Moreover, we investigated the factors affecting the peroxidase-like activity of SiO_2_@Au NPs, such as TMB concentration, H_2_O_2_ concentration, pH, SiO_2_@Au NPs amount, reaction time, and termination time. Furthermore, SiO_2_@Au NPs showed high stability during the 30-day-long storage time at room temperature and outstanding reusability for five cycles. This work is therefore meaningful for utilizing controllable nanoparticles in various fields and provides a better approach to develop nanoparticle-based nanozymes.

## Figures and Tables

**Table 1:** Amount of material controlling TEM) images of gold-embedded silica nanospheres (SiO_2_@Au NPs) and its effect. The sizes of Au NPs were measured by using TEM images.

Sample	Au-Seeded SiO_2_ (mg)	Au^3+^ (μM)	Ascorbic Acid (μM)	Au Size (nm)	λ_max_ (nm)	λ_max_ (a.u.)	Suspension Color
I	200	0	0	1.41	-	-	Pale brown
Ii	200	50	100	4.33	543	0.27	Pink
Iii	200	100	200	6.42	571	0.54	Purple
Iv	200	150	300	7.33	593	0.87	Dark blue
V	200	200	400	9.56	619	1.20	Dark blue
Vi	200	300	600	15.27	632	1.27	Dark blue

Sample numbers correspond to those mentioned in Figure 1.

## Data Availability

Data is contained within the article or supplementary material.

## Supplementary Materials

The following are available online at https://www.mdpi.com/article/10.3390/ijms221910382/s1.

## References

[1] Wei H., Wang E. (2013). Nanomaterials with enzyme-like characteristics (nanozymes): Next-generation artificial enzymes. Chem. Soc. Rev..

[2] Manea F., Houillon F.B., Pasquato L., Scrimin P. (2004). Nanozymes: Gold-Nanoparticle-Based Transphosphorylation Catalysts. Angew. Chem. Int. Ed..

[3] Wei H., Wang E. (2008). Fe3O4 Magnetic Nanoparticles as Peroxidase Mimetics and Their Applications in H2O2 and Glucose Detection. Anal. Chem..

[4] Antuña-Jiménez D., Blanco-López M.C., Miranda-Ordieres A.J., Lobo-Castañón M.J. (2014). Artificial enzyme with magnetic properties and peroxidase activity on indoleamine metabolite tumor marker. Polymers.

[5] Zhang K., Hu X., Liu J., Yin J.-J., Hou S., Wen T., He W., Ji Y., Guo Y., Wang Q. (2011). Formation of PdPt Alloy Nanodots on Gold Nanorods: Tuning Oxidase-like Activities via Composition. Langmuir.

[6] He W., Jia H., Li X., Lei Y., Li J., Zhao H., Mi L., Zhang L., Zheng Z. (2012). Understanding the formation of CuS concave superstructures with peroxidase-like activity. Nanoscale.

[7] Ju H. (2011). Sensitive biosensing strategy based on functional nanomaterials. Sci. China Ser. B Chem..

[8] Celardo I., Pedersen J.Z., Traversa E., Ghibelli L. (2011). Pharmacological potential of cerium oxide nanoparticles. Nanoscale.

[9] Kotov N.A. (2010). Inorganic Nanoparticles as Protein Mimics. Science.

[10] Gao L., Zhuang J., Nie L., Zhang J., Zhang Y., Gu N., Wang T., Feng J., Yang D., Perrett S. (2007). Intrinsic peroxidase-like activity of ferromagnetic nanoparticles. Nat. Nanotechnol..

[11] Cortie M.B., van der Lingen E. (2002). Catalytic Gold Nano-Particles. Mater. Forum.

[12] Bond G.C. (2001). Gold: A relatively new catalyst. Gold Bull..

[13] Asati A., Santra S., Kaittanis C., Nath S., Perez J.M. (2009). Oxidase-Like Activity of Polymer-Coated Cerium Oxide Nanoparticles. Angew. Chem. Int. Ed..

[14] Chen W., Chen J., Feng Y.-B., Hong L., Chen Q.-Y., Wu L.-F., Lin X.-H., Xia X.-H. (2012). Peroxidase-like activity of water-soluble cupric oxide nanoparticles and its analytical application for detection of hydrogen peroxide and glucose. Analyst.

[15] Wang J., Zhao H., Song J., Zhu T., Xu W. (2019). Structure-Activity Relationship of Manganese Oxide Catalysts for the Catalytic Oxidation of (chloro)-VOCs. Catalysts.

[16] Karakoti A., Singh S., Dowding J.M., Seal S., Self W. (2010). Redox-active radical scavenging nanomaterials. Chem. Soc. Rev..

[17] Jiao X., Song H., Zhao H., Bai W., Zhang L., Lv Y. (2012). Well-redispersed ceria nanoparticles: Promising peroxidase mimetics for H2O2 and glucose detection. Anal. Methods.

[18] Zhang X.-Q., Gong S.-W., Zhang Y., Yang T., Wang C.-Y., Gu N. (2010). Prussian blue modified iron oxide magnetic nanoparticles and their high peroxidase-like activity. J. Mater. Chem..

[19] Chaudhari K.N., Chaudhari N., Yu J.-S. (2012). Peroxidase mimic activity of hematiteiron oxides (α-Fe2O3) with different nanostructures. Catal. Sci. Technol..

[20] Dutta A.K., Maji S.K., Srivastava D.N., Mondal A., Biswas P., Paul P., Adhikary B. (2012). Peroxidase-like activity and amperometric sensing of hydrogen peroxide by Fe2O3 and Prussian Blue-modified Fe2O3 nanoparticles. J. Mol. Catal. A Chem..

[21] Comotti M., Della Pina C., Falletta E., Rossi M. (2006). Aerobic Oxidation of Glucose with Gold Catalyst: Hydrogen Peroxide as Intermediate and Reagent. Adv. Synth. Catal..

[22] Shen X., Liu W., Gao X., Lu Z., Wu X., Gao X. (2015). Mechanisms of Oxidase and Superoxide Dismutation-like Activities of Gold, Silver, Platinum, and Palladium, and Their Alloys: A General Way to the Activation of Molecular Oxygen. J. Am. Chem. Soc..

[23] Medley C.D., Smith J.E., Tang Z., Wu Y., Bamrungsap S., Tan W. (2008). Gold Nanoparticle-Based Colorimetric Assay for the Direct Detection of Cancerous Cells. Anal. Chem..

[24] Popovtzer R., Agrawal A., Kotov N., Popovtzer A., Balter J., Carey T., Kopelman R. (2008). Targeted Gold Nanoparticles Enable Molecular CT Imaging of Cancer. Nano Lett..

[25] Fang S.-B., Tseng W.Y., Lee H.-C., Tsai C.-K., Huang J.-T., Hou S.-Y. (2009). Identification of Salmonella using colony-print and detection with antibody-coated gold nanoparticles. J. Microbiol. Methods.

[26] Kim C.S., Wilder-Smith P., Ahn Y.-C., Liaw L.-H.L., Chen Z., Kwon Y.J. (2009). Enhanced detection of early-stage oral cancer in vivo by optical coherence tomography using multimodal delivery of gold nanoparticles. J. Biomed. Opt..

[27] Thaxton C.S., Elghanian R., Thomas A.D., Stoeva S.I., Lee J.-S., Smith N.D., Schaeffer A.J., Klocker H., Horninger W., Bartsch G. (2009). Nanoparticle-based bio-barcode assay redefines "undetectable" PSA and biochemical recurrence after radical prostatectomy. Proc. Natl. Acad. Sci. USA.

[28] Zhang J., Wang L., Zhang H., Boey F., Song S., Fan C. (2010). Aptamer-Based Multicolor Fluorescent Gold Nanoprobes for Multiplex Detection in Homogeneous Solution. Small.

[29] Huo Q., Colon J., Cordero A., Bogdanovic J., Baker C.H., Goodison S., Pensky M.Y. (2011). A Facile Nanoparticle Immunoassay for Cancer Biomarker Discovery. J. Nanobiotechnol..

[30] LeDuc C., Jung J.-M., Carney R.R., Stellacci F., Lounis B. (2011). Direct Investigation of Intracellular Presence of Gold Nanoparticles via Photothermal Heterodyne Imaging. ACS Nano.

[31] Von Maltzahn G., Park J.-H., Lin K.Y., Singh N., Schwöppe C., Mesters R., Berdel W.E., Ruoslahti E., Sailor M.J., Bhatia S.N. (2011). Nanoparticles that communicate in vivo to amplify tumour targeting. Nat. Mater..

[32] Wang H., Zheng L., Peng C., Guo R., Shen M., Shi X., Zhang G. (2011). Computed tomography imaging of cancer cells using acetylated dendrimer-entrapped gold nanoparticles. Biomaterials.

[33] Zhang Y., Qian J., Wang D., Wang Y., He S. (2012). Multifunctional Gold Nanorods with Ultrahigh Stability and Tunability for In Vivo Fluorescence Imaging, SERS Detection, and Photodynamic Therapy. Angew. Chem. Int. Ed..

[34] Youssef A.M., Abdel-Aziz M., El-Sayed S. (2014). Chitosan nanocomposite films based on Ag-NP and Au-NP biosynthesis by Bacillus Subtilis as packaging materials. Int. J. Biol. Macromol..

[35] Zhang Z., Wang J., Nie X., Wen T., Ji Y., Wu X., Zhao Y., Chen C. (2014). Near Infrared Laser-Induced Targeted Cancer Therapy Using Thermoresponsive Polymer Encapsulated Gold Nanorods. J. Am. Chem. Soc..

[36] Grzelczak M., Pérez-Juste J., Mulvaney P., Liz-Marzán L.M. (2008). Shape control in gold nanoparticle synthesis. Chem. Soc. Rev..

[37] Daniel M.-C., Astruc D. (2004). Gold Nanoparticles: Assembly, Supramolecular Chemistry, Quantum-Size-Related Properties, and Applications toward Biology, Catalysis, and Nanotechnology. Chem. Rev..

[38] Henglein A. (1993). Physicochemical properties of small metal particles in solution: "Microelectrode" reactions, chemisorption, composite metal particles, and the atom-to-metal transition. J. Phys. Chem..

[39] Belloni J. (1996). Metal nanocolloids. Curr. Opin. Colloid Interface Sci..

[40] Toshima N., Yonezawa T. (1998). Bimetallic nanoparticles—novel materials for chemical and physical applications. New J. Chem..

[41] Brust M., Kiely C. (2002). Some recent advances in nanostructure preparation from gold and silver particles: A short topical review. Colloids Surf. A Physicochem. Eng. Asp..

[42] Lin Y.-C., Yu B.-Y., Lin W.-C., Lee S.-H., Kuo C.-H., Shyue J.-J. (2009). Tailoring the surface potential of gold nanoparticles with self-assembled monolayers with mixed functional groups. J. Colloid Interface Sci..

[43] Fan K., Cao C., Pan Y., Lu D., Yang D., Feng J., Song L., Liang M., Yan X. (2012). Magneto ferritin nanoparticles for targeting and visualizing tumour tissues. Nat. Nanotechnol..

[44] He W., Zhou Y.-T., Wamer W.G., Hu X., Wu X., Zheng Z., Boudreau M.D., Yin J.-J. (2013). Intrinsic catalytic activity of Au nanoparticles with respect to hydrogen peroxide decomposition and superoxide scavenging. Biomaterials.

[45] Westcott S.L., Oldenburg S.J., Lee A.T.R., Halas N. (1998). Formation and Adsorption of Clusters of Gold Nanoparticles onto Functionalized Silica Nanoparticle Surfaces. Langmuir.

[46] Prodan E., Nordlander P., Halas N.J. (2003). Electronic Structure and Optical Properties of Gold Nanoshells. Nano Lett..

[47] Wilhelm P., Stephan D. (2006). On-line tracking of the coating of nanoscaled silica with titania nanoparticles via zeta-potential measurements. J. Colloid Interface Sci..

[48] Loo C., Lin A., Hirsch L., Lee M.-H., Barton J., Halas N., West J., Drezek R. (2004). Nanoshell-Enabled Photonics-Based Imaging and Therapy of Cancer. Technol. Cancer Res. Treat..

[49] Zhang Y.-F., Wang J.-H., Ma L., Nan F., Cheng Z.-Q., Zhou L., Wang Q.-Q. (2015). Growth of silver-coated gold nanoshells with enhanced linear and nonlinear optical responses. J. Nanoparticle Res..

[50] Lu L., Zhang H., Sun G., Xi A.S., Wang H., Li X., Wang A.X., Zhao B. (2003). Aggregation-Based Fabrication and Assembly of Roughened Composite Metallic Nanoshells: Application in Surface-Enhanced Raman Scattering. Langmuir.

[51] Gawande M.B., Goswami A., Asefa T., Guo H., Biradar A.V., Peng D.-L., Zboril R., Varma R.S. (2015). Core–shell nanoparticles: Synthesis and applications in catalysis and electrocatalysis. Chem. Soc. Rev..

[52] Xue J., Wang C., Ma Z. (2007). A facile method to prepare a series of SiO2@Au core/shell structured nanoparticles. Mater. Chem. Phys..

[53] Brito-Silva A.M., Sobral-Filho R.G., Barbosa-Silva R., de Araújo C.B., Galembeck A., Brolo A.G. (2013). Improved Synthesis of Gold and Silver Nanoshells. Langmuir.

[54] Tharion J., Satija J., Mukherji S. (2014). Glucose mediated synthesis of gold nanoshells: A facile and eco-friendly approach conferring high colloidal stability. RSC Adv..

[55] Garcia-Soto M.J., González-Ortega O. (2016). Synthesis of silica-core gold nanoshells and some modifications/variations. Gold Bull..

[56] Shim S., Pham X.-H., Cha M.G., Lee Y.-S., Jeong D.H., Jun B.-H. (2016). Size effect of gold on Ag-coated Au nanoparticle-embedded silica nanospheres. RSC Adv..

[57] Pham X.-H., Hahm E., Kang E., Na Ha Y., Lee S.H., Rho W.-Y., Lee Y.-S., Jeong D.H., Jun B.-H. (2019). Gold-silver bimetallic nanoparticles with a Raman labeling chemical assembled on silica nanoparticles as an internal-standard-containing nanoprobe. J. Alloy. Compd..

[58] Su K.-H., Wei A.Q.-H., Zhang X., Mock J.J., Smith A.D.R., Schultz S. (2003). Interparticle Coupling Effects on Plasmon Resonances of Nanogold Particles. Nano Lett..

[59] Jain P., El-Sayed M.A. (2010). Plasmonic coupling in noble metal nanostructures. Chem. Phys. Lett..

[60] Seong B., Bock S., Hahm E., Huynh K.-H., Kim J., Lee S.H., Pham X.-H., Jun B.-H. (2021). Synthesis of Densely Immobilized Gold-Assembled Silica Nanostructures. Int. J. Mol. Sci..

[61] Pham X.-H., Hahm E., Kang E., Son B.S., Ha Y., Kim H.-M., Jeong D.H., Jun B.-H. (2019). Control of Silver Coating on Raman Label Incorporated Gold Nanoparticles Assembled Silica Nanoparticles. Int. J. Mol. Sci..

[62] Pham X.-H., Hahm E., Huynh K.-H., Son B.S., Kim H.-M., Jeong D.H., Jun B.-H. (2019). 4-Mercaptobenzoic Acid Labeled Gold-Silver-Alloy-Embedded Silica Nanoparticles as an Internal Standard Containing Nanostructures for Sensitive Quantitative Thiram Detection. Int. J. Mol. Sci..

[63] Link S., El-Sayed M.A. (1999). Spectral Properties and Relaxation Dynamics of Surface Plasmon Electronic Oscillations in Gold and Silver Nanodots and Nanorods. J. Phys. Chem. B.

[64] Josephy P.D., Eling T., Mason R.P. (1982). The horseradish peroxidase-catalyzed oxidation of 3,5,3’,5’-tetramethylbenzidine. Free radical and charge-transfer complex intermediates. J. Biol. Chem..

[65] Li B.L., Luo H.Q., Lei J.L., Li N.B. (2014). Hemin-functionalized MoS2 nanosheets: Enhanced peroxidase-like catalytic activity with a steady state in aqueous solution. RSC Adv..

[66] Ma M., Zhang Y., Gu N. (2011). Peroxidase-like catalytic activity of cubic Pt nanocrystals. Colloids Surf. A Physicochem. Eng. Asp..

[67] Asati A., Kaittanis C., Santra S., Perez J.M. (2011). pH-Tunable Oxidase-Like Activity of Cerium Oxide Nanoparticles Achieving Sensitive Fluorigenic Detection of Cancer Biomarkers at Neutral pH. Anal. Chem..

[68] Ge C., Fang G., Shen X., Chong Y., Wamer W.G., Gao X., Chai Z., Chen C., Yin J.-J. (2016). Facet Energy versus Enzyme-like Activities: The Unexpected Protection of Palladium Nanocrystals against Oxidative Damage. ACS Nano.

[69] Lin L., Song X., Chen Y., Rong M., Zhao T., Wang Y., Jiang Y., Chen X. (2015). Intrinsic peroxidase-like catalytic activity of nitrogen-doped graphene quantum dots and their application in the colorimetric detection of H2O2 and glucose. Anal. Chim. Acta.

[70] Shah V., Shah S., Shah H., Rispoli F.J., McDonnell K.T., Workeneh S., Karakoti A., Kumar A., Seal S. (2012). Antibacterial Activity of Polymer Coated Cerium Oxide Nanoparticles. PLoS ONE.

[71] Frey A., Meckelein B., Externest D., Schmidt M. (2000). A stable and highly sensitive 3,3′,5,5′-tetramethylbenzidine-based substrate reagent for enzyme-linked immunosorbent assays. J. Immunol. Methods.

[72] Jiang B., Duan D., Gao L., Zhou M., Fan K., Tang Y., Xi J., Bi Y., Tong Z., Gao G.F. (2018). Standardized assays for determining the catalytic activity and kinetics of peroxidase-like nanozymes. Nat. Protoc..

[73] Gökçal B., Hamaloğlu K.Ö., Kip Ç., Güngör S.Y., Büber E., Tuncel A. (2020). Glutathione detection in human serum using gold nanoparticle decorated, monodisperse porous silica microspheres in the magnetic form. Anal. Methods.

[74] Gökçal B., Kip Ç., Tuncel A. (2020). One-pot, direct glucose detection in human whole blood without using a dilution factor by a magnetic nanozyme with dual enzymatic activity. J. Alloy. Compd..

[75] Alsharif N.B., Bere K., Sáringer S., Samu G.F., Takács D., Hornok V., Szilagyi I. (2021). Design of hybrid biocatalysts by controlled heteroaggregation of manganese oxide and sulfate latex particles to combat reactive oxygen species. J. Mater. Chem. B.

[76] Alsharif N.B., Samu G.F., Sáringer S., Muráth S., Szilagyi I. (2020). A colloid approach to decorate latex particles with Prussian blue nanozymes. J. Mol. Liq..

[77] Tian J., Liu S., Luo Y., Sun X. (2012). Fe(III)-based coordination polymer nanoparticles: Peroxidase-like catalytic activity and their application to hydrogen peroxide and glucose detection. Catal. Sci. Technol..

[78] Song Y., Qu K., Zhao C., Ren J., Qu X. (2010). Graphene Oxide: Intrinsic Peroxidase Catalytic Activity and Its Application to Glucose Detection. Adv. Mater..

[79] Lipinski B. (2011). Hydroxyl Radical and Its Scavengers in Health and Disease. Oxidative Med. Cell. Longev..

[80] You L., Mao Y., Ge J. (2012). Synthesis of Stable SiO2@Au-Nanoring Colloids as Recyclable Catalysts: Galvanic Replacement Taking Place on the Surface. J. Phys. Chem. C.

