# Supplementary material for: Synthesis of Finely Controllable Sizes of Au Nanoparticles on a Silica Template and Their Nanozyme Properties
Source: Int J Mol Sci. 2021 Sep 26;22(19):10382. doi: 10.3390/ijms221910382 (PMC8508978; doi:10.3390/ijms221910382)
Supplement: Supplementary file 1 [file ijms-22-10382-s001.zip › ijms-1373201-supplementary.pdf]

Synthesis of Finely Controllable Sizes of Au Nanoparticles on a Silica Template and Their Nanozyme Properties

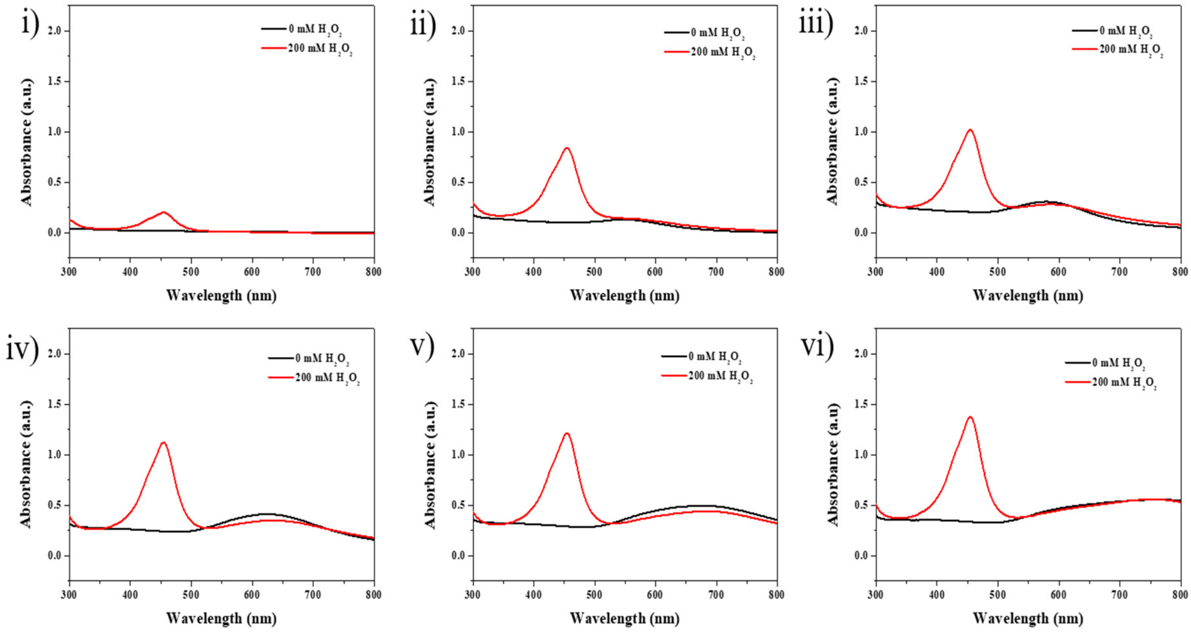

Figure S1. UV-Vis spectra of 0.1 mM TMB and 200 mM H_2O_2 in the presence of various SiO_2@Au NPs fabricated at (i) 0 μM, (ii) 50 μM, (iii) 100 μM, (iv) 150 μM, (v) 200 μM, and (vi) 300 μM of Au^{3+} concentration.

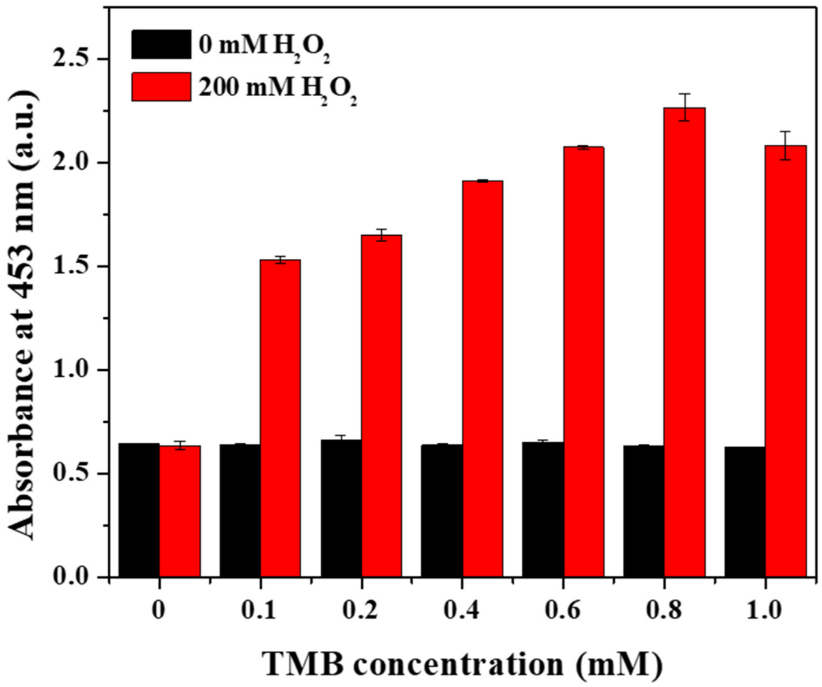

Figure S2. The effects of TMB concentration in the range of 0 to 1.0 mM on the peroxidase-like activity of SiO_2@Au in mixture of TMB and 200 mM H_2O_2: The UV-Vis absorbance plots of each condition at 453 nm wavelength which is absorbance of oxidated TMB substrate.

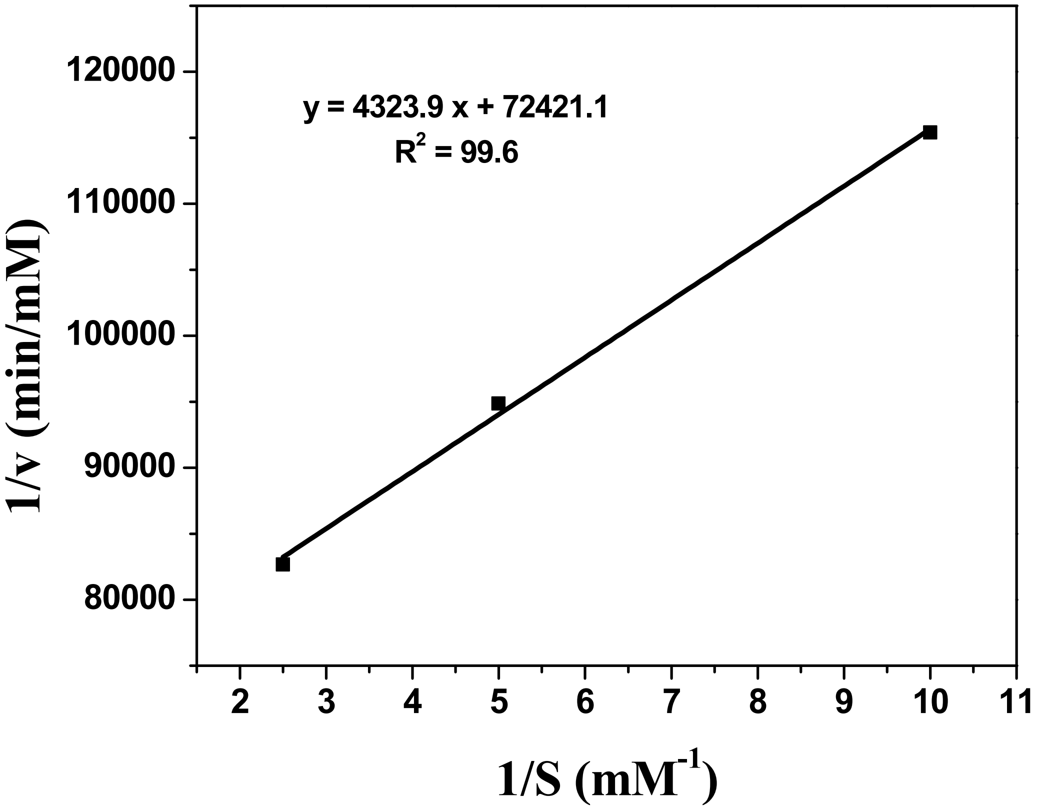

Figure S3. Kinetic analysis of SiO_2@Au. Kinetic parameters of SiO_2@Au were determined by using various TMB concentrations of 0.1-0.4 mM. The value of Michaelis constant K_m and maximal velocity of the reaction V_{max} is attained according to the Lineweaver-Burk equation (1/v=Km/(Vmax*[S]) + 1/Vmax).

$V_{max} = 2.3 \times 10^{-10}$ M.s^{-1}

$K_m = 0.0597$ mM

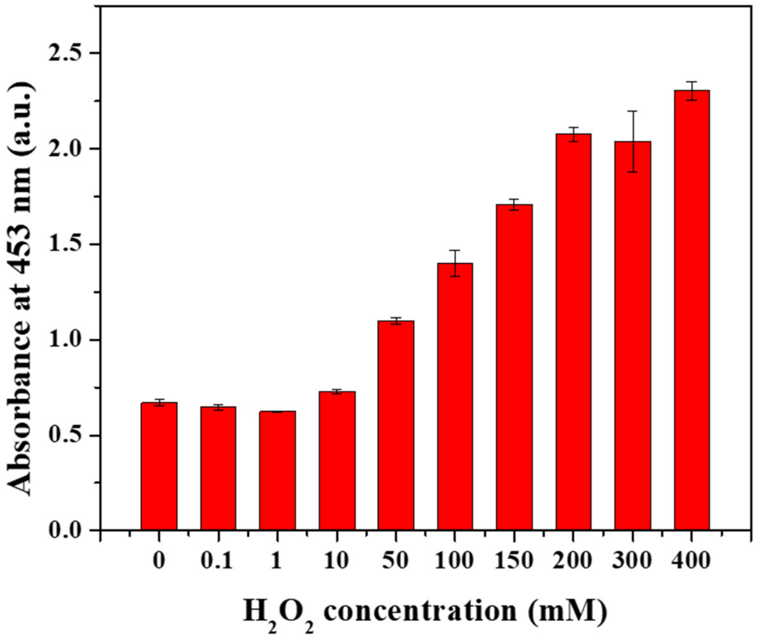

Figure S4. The effects of H_2O_2 concentration in the range of 0.1 to 400 mM on the peroxidase-like activity of SiO_2@Au in mixture of 0.6 mM TMB and H_2O_2. The UV-Vis absorbance plots of each condition at 453 nm wavelength which is absorbance of oxidated TMB substrate.

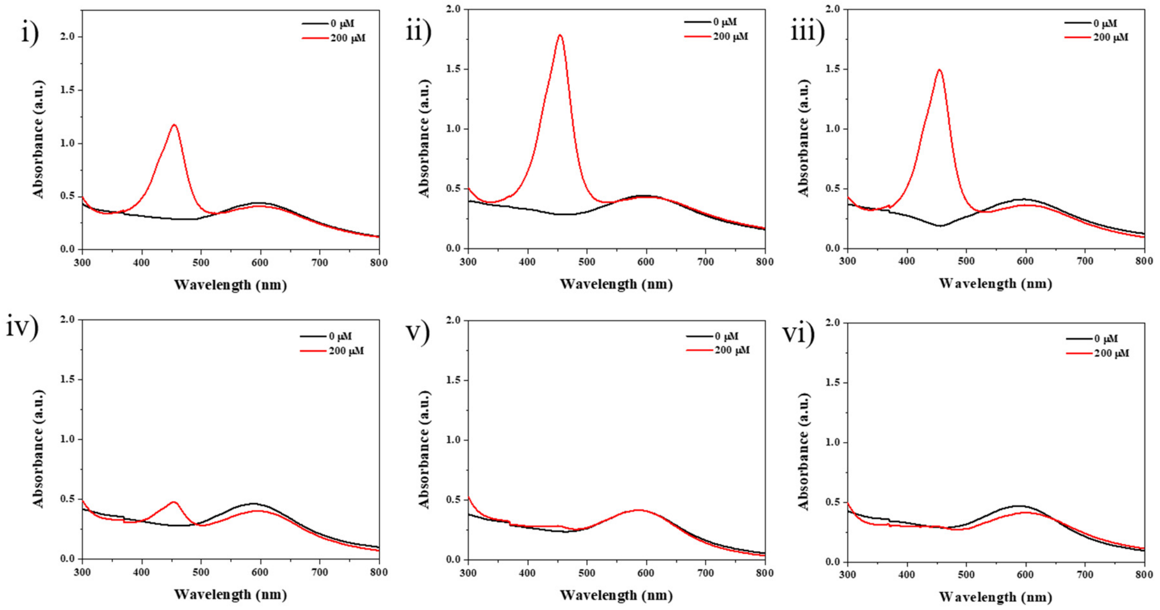

Figure S5. Effect of pH solution on the UV-Vis spectra of 0.6 mM TMB and 200 mM H_2O_2 in the presence of SiO_2@Au at various pH conditions: (i) pH 3.0, (ii) pH 4.0, (iii) pH 5.0, (iv) pH 6.0, (v) pH 7.0, and (vi) pH 8.0.

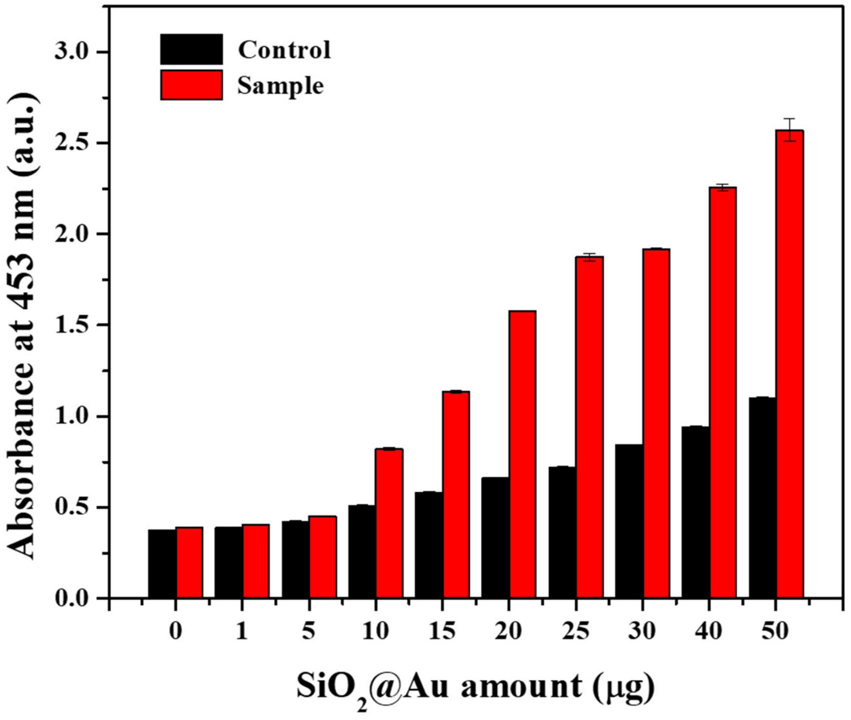

Figure S6. The effects of SiO_2@Au amount in the range of 0 to 50 μg on the peroxidase-like activity of SiO_2@Au in mixture of 0.6 mM TMB and H_2O_2. The UV-Vis absorbance plots of each condition at 453 nm wavelength which is absorbance of oxidated TMB substrate.

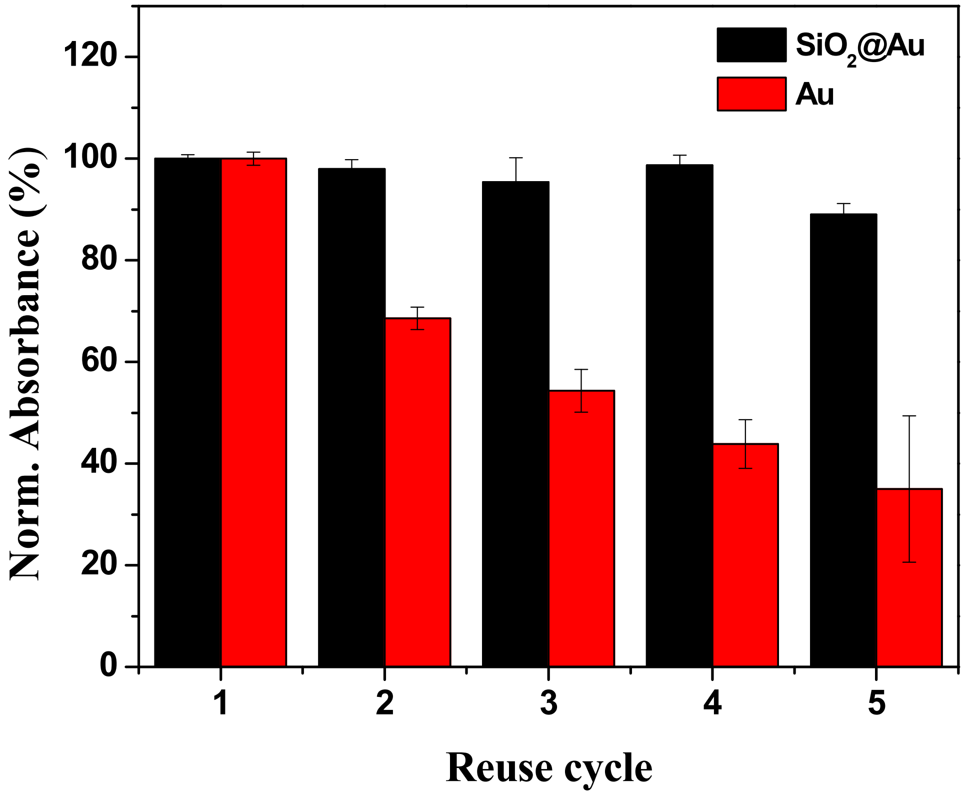

Figure S7. Reusability of SiO_2@Au and Au NPs in a mixture of 0.6 mM TMB, 200 mM H_2O_2, and pH 4 buffer. The SiO_2@Au and Au NPs were stored in PBST (0.1%) at room temperature.
